# Supplementary material for: Correlation of Transcriptomics and FDG-PET SUVmax Indicates Reciprocal Expression of Stemness-Related Transcription Factor and Neuropeptide Signaling Pathways in Glucose Metabolism of Ewing Sarcoma
Source: Cancers (Basel). 2022 Dec 5;14(23):5999. doi: 10.3390/cancers14235999 (PMC9735504; doi:10.3390/cancers14235999)

Suppl. Figures

Suppl. Figure S1: Survival analysis. (a) Kaplan-Meier curves for univariate analyses. There is no significant difference in overall survival with regard to sex (male vs. female), disease state (primary disease vs. relapse), age (≤15 years vs. >15 years), or SUV categories (low SUV vs. high SUV). (b) Forest plot for multivariate Cox proportional hazards model including continuous SUVmax and disease state (primary disease vs. relapse). Higher SUVmax and relapse are significantly associated with increased risk of death (global p-value=0.02 in log-rank test). For continuous SUVmax, the hazard ratio is 1.2 per increase of 1 SUV unit.

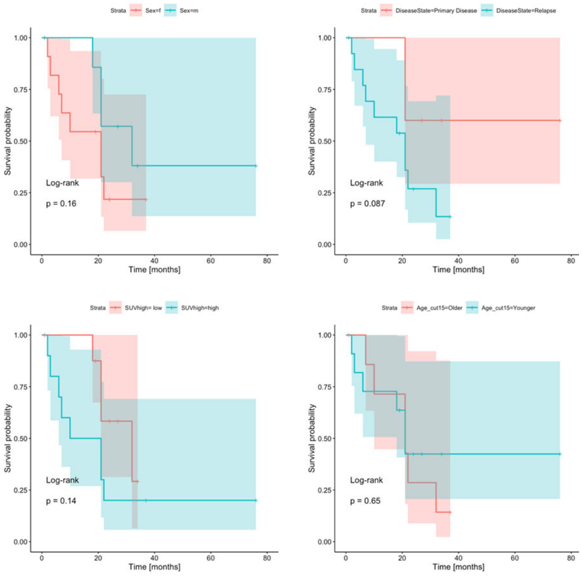

(a)

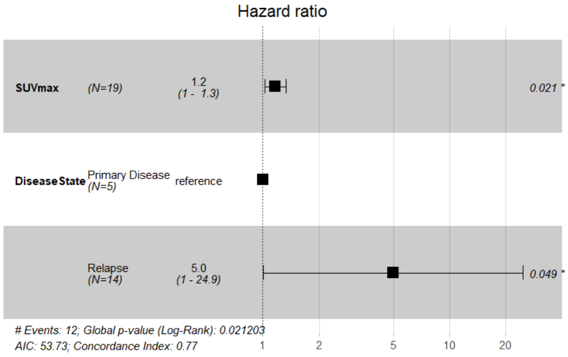

(b)

Suppl. Figure S2. Volcano plot showing results from linear regression modeling of SUVmax and gene expression. For each gene tested, the slope of the regression line and significance in form of -log10(adjusted p-value) are given. 5 genes are significantly correlated with SUVmax with adjusted p-value<0.05 (FAXDC2, MYBL2, PLSCR4, ELOVL2, and NETO2). 23 genes show a high effect size of abs(slope)>0.146 (labelled genes).

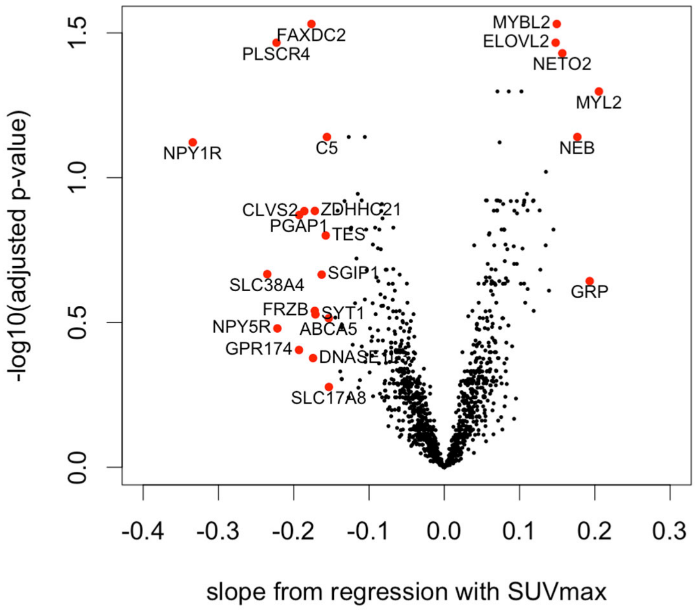

Suppl. Figure S3. Correlation of SUVmax and the NPY axis. Expression of members of the NPY axis (NPY, NPY1R, NPY5R) decreases with increasing SUVmax.

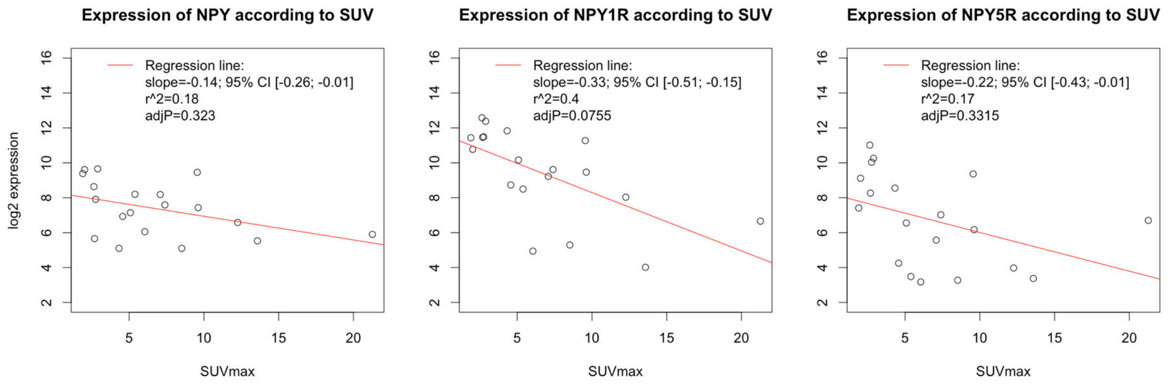

Supplement: Supplementary file 1 [file cancers-14-05999-s001.zip › SupplFigures.pdf]
